# Forearm and elbow secondary surgical procedures in neonatal brachial plexus palsy: a systematic scoping review

**DOI:** 10.1016/j.xrrt.2023.10.003

**Published:** 2023-11-15

**Authors:** Amanda Azer, Aedan Hanna, Dhvani Shihora, Anthony Saad, Yajie Duan, Aleksandra McGrath, Alice Chu

**Affiliations:** a Department of Orthopaedic Surgery, Rutgers New Jersey Medical School, Newark, NJ, USA; b Department of Statistics, Rutgers University, Newark, NJ, USA; c Department of Clinical Sciences, Umeå- University, Umeå, Sweden; d Department of Surgical and Perioperative Sciences, Umeå University, Umeå, Sweden; e Department of Orthopedic Surgery, Rutgers University, Newark, NJ, USA

**Keywords:** Neonatal brachial plexus palsy, Secondary surgery, Forearm surgery, Elbow surgery, Brachial plexus, Surgical outcomes

## Abstract

**Background:**

Neonatal Brachial plexus palsy is an injury during delivery that can lead to loss of motor function and limited range of motion in patients due to damage of nerves in the brachial plexus. This scoping review aims to explore types of procedures performed and assess outcomes of forearm and elbow secondary surgery in pediatric patients.

**Methods:**

Searches of PubMed, Cochrane, Cumulative Index to Nursing and Allied Health Literature, Web of Sciences, and Scopus were completed to obtain studies describing surgical treatment of elbow and forearm in pediatric patients with neonatal Brachial plexus palsy. 865 abstracts and titles were screened by two independent reviewers resulting in 295 full text papers; after applying of inclusion and exclusion criteria 18 articles were included. The level of evidence of this study is level IV.

**Results:**

Ten main procedures were performed to regain function of the forearm and elbow in neonatal brachial plexus birth palsy patients. Procedures had different aims, with supination contracture (6) and elbow flexion restoration (5) being the most prevalent. The variance between preoperative and postoperative soft tissue and bony procedures outcomes decreased and showed improvement with respect to the aim of each procedure category. For soft tissue procedures, a statistically significant increase was found between preoperative and postoperative values for active elbow flexion, passive supination, and active supination. For bony procedures, there was a statistically significant decrease between preoperative and postoperative values of passive and active supination.

**Conclusion:**

Overall, all procedures completed in the assessed articles of this study were successful in their aim. Bony procedures, specifically osteotomies, were found to have a wider range of results, whereas soft tissue procedures were found to be more consistent and reproducible with respect to their outcomes. Bony and soft tissue procedures were found vary in their aims and outcomes. This study indicates the need for further research to augment knowledge about indications and long-term benefits to each procedure.

Neonatal brachial plexus palsy’s (NBPP’s) prevalence has remained stable affecting about 0.05%-0.4% of live births.^1^^,^^21^^,^^45^ Recovery to normal function among those who do not need primary surgery is generally high, ranging from 66%-92%.^11^^,^^39^ However, between 14%-62% of patients have residual disability, which could necessitate secondary surgery.^13^^,^^23^^,^^47^ Residual disability depends on the muscle or joint affected along with whether it is a global, upper, or lower plexus palsy.^11^ Secondary surgery is indicated for patient who failed to recover function in the upper limbs either spontaneously or after primary nerve surgery.^1^^,^^41^

The most common functional deformities occur in the shoulder, elbow, forearm, wrist, and hand. The shoulder is the most affected of the above; however, the forearm and elbow are also impacted leading to a range of motion losses including pronation, supination, flexion, and extension. Shoulder secondary procedures represent the majority of secondary surgeries performed on NBPP patients and are well described in the literature.^9^^,^^32^^,^^37^^,^^36^ Procedures specific to the elbow and forearm are less represented in the literature, reflecting likely lower volumes of patients needing these particular surgeries. Forearm and elbow deformities include pronation contracture, supination contracture and flexion contracture with limited elbow extension. Elbow involvement usually presents as a flexion contracture.^1^^,^^41^ Within the population of brachial plexus birth injury patients, the median prevalence of an elbow flexion contracture is 48%.^17^^,^^45^

Currently, there is a lack of literature that explores studies describing the varying procedures for NBPP patients, which could be used to guide surgical decision making. The aim of this systematic scoping review is to catalog and compare the types of secondary procedures for functional restoration of elbow and forearm deformities, their timing, and their outcomes.

## Materials and methods

### Literature search

**Figure 1:**
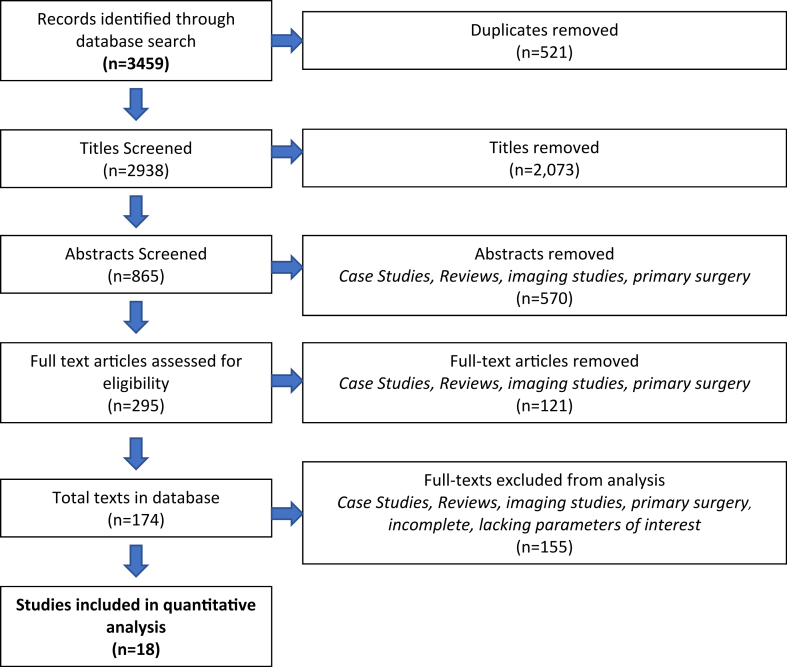
The PRISMA flowchart is depicting the search strategy for this study is presented above. *PRISMA*, Preferred Reporting Items for Systematic Review and Meta-Analyses.

Following Preferred Reporting Items for Systematic Review and Meta-Analyses-Scoping Review (PRISMA-ScR) guidelines, a search was conducted of PubMed, Cochrane, Cumulative Index to Nursing and Allied Health Literature, Web of Sciences, and Scopus on NBPP.^46^ Inclusion criteria was comprised of original articles, including randomized control trials, review papers and retrospective cohort studies, that described secondary surgical procedures on the forearm or elbow following NBPP. Only articles that documented patients less than 18 years of age at the time of the procedure, and articles were required to have a minimum of 3 patients. Exclusion criteria and PRISMA flowchart can be found in Figure 1. The initial review was done by three independent reviewers (AA, DS, AH), and any discrepancies were resolved independently by the principal investigators (AC, AM).

### Data extraction and screening

Data points collected from each study included year of publication, number of patients, average patient age, patient sex, average follow-up, treatment or procedure completed, aim of the study, and all outcome measures presented by each article. Outcome measures included forearm supination and pronation, elbow flexion and extension, wrist motion variables, Raimondi score for hand function, British Medical Research Council score, and biceps strength. Both itemized and mean data points were collected depending on what the article presented. Studies were grouped into bony, soft tissue, and botulinum toxin procedures. For bias assessment, Robins I-Risk of Bias in Non-Randomized Studies-of Interventions (ROBINS-I) was used.^4^

### Statistical analysis

All statistical analysis was completed using itemized data collected. Paired t tests and paired Wilcoxon tests were run on the itemized data with a level of significance at a α = 0.01 to determine if there were significant differences in ages of patients undergoing procedures, follow-up time, and preoperative and postoperative outcomes within each subgroup. An unpaired Wilcoxon test was completed to compare the preoperative and postoperative outcome values between the two subgroups. A value of *P* < .05 was considered statistically significant. To compare the distributions of preoperative and postoperative outcomes of bony vs. soft tissue, a Kolmogorov-Smirnov (KS) test was used. Statistical analysis was not completed on the mean data values as overlapping outcome measures were too few to yield significant results.

## Results

### Review characteristics

Three thousand four hundred fifty-nine records were obtained and of those 2938 titles were included following the removal of duplicates. 865 abstracts were screened by two independent reviewers. 295 full text papers were then screened by two reviewers applying inclusion and exclusion criteria. 18 full text papers were included in this review; see the PRISMA flowchart (Fig. 1).^29^ As no time limits were set, studies published between 1964 and 2021 were included.

### Risk of bias and quality of evidence

**Figure 2:**
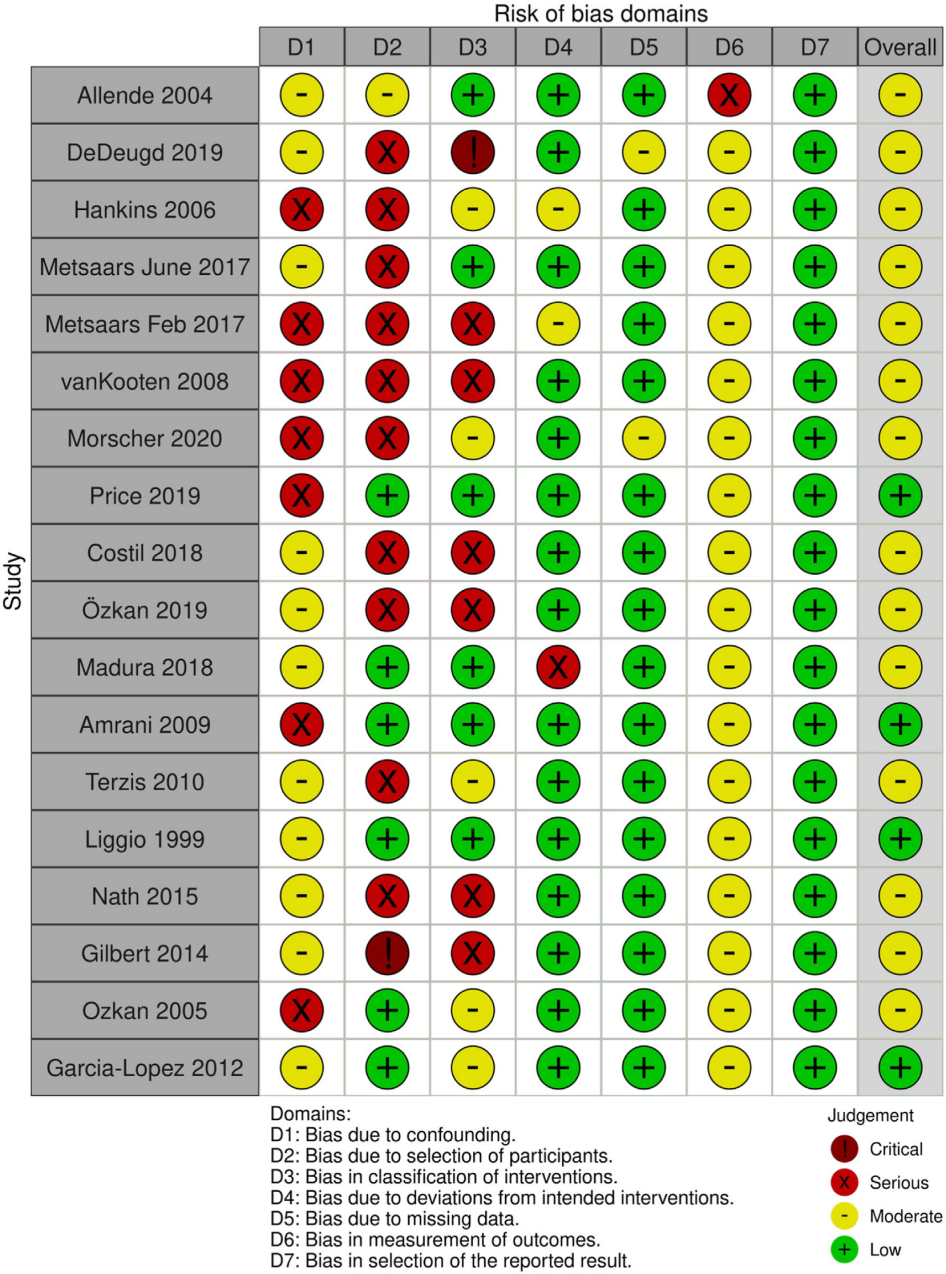
ROBINS-I risk of bias assessment. *ROBINS-I*, Robins I-Risk of Bias in Non-Randomized Studies-of Interventions.

The risk of bias was assessed using the ROBINS-I tool for nonrandomized studies. Of these manuscripts, 14 had moderate bias and 4 had low bias. None of the manuscripts had severe or critical bias. Because all the studies had low number of participants, all studies scored low for selection of the reported result. Several manuscripts also had low bias due complete data sets. All studies had some bias due to confounding factors, receiving either a moderate or severe bias score. According to the ROBINS-I table, moderate bias was the overall score for all studies combined (Fig. 2).^42^

### Outcomes and interventions

**Figure 3:**
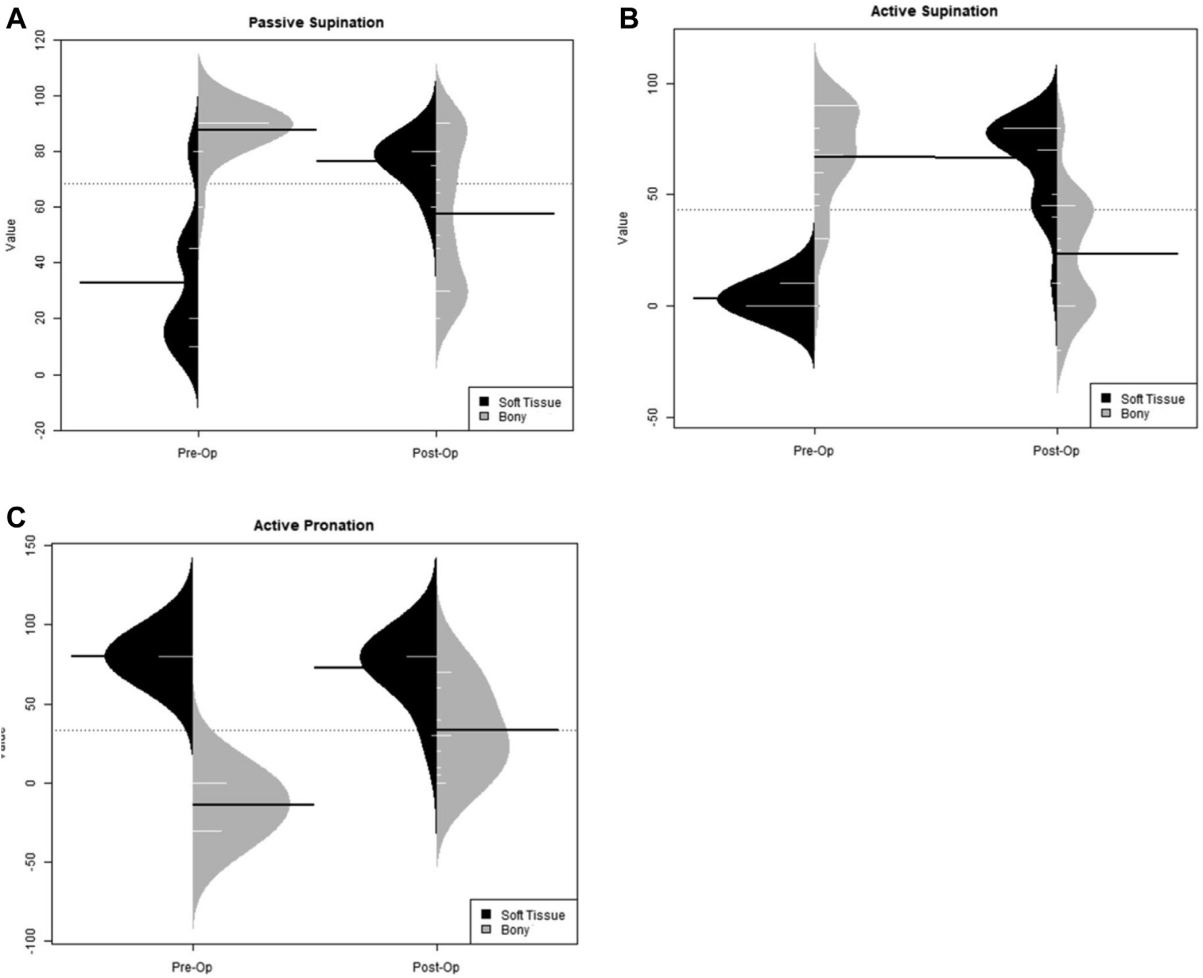
Preoperative and Postoperative comparison of distributions several of range of motion outcomes are presented above. This represents the differences found between bony and soft tissue procedures and within each group preoperative and postoperatively. The green group represents soft tissue procedures’ outcomes, and the orange group represents the bony procedures’ outcome distributions. (**A**) describes the preoperative and postoperative changes in distribution and degrees for both bony and soft tissue procedures for passive supination. Soft tissue procedures had a reduced variance with an increase in degrees of supination, whereas bony procedures had the opposite. (**B**) describes the same for active supination. Soft tissue procedures had a reduced variance with an increase in degrees of supination, whereas bony procedures had the opposite. (**C**) does the same for active pronation. Soft tissue procedures showed similar variance with similar degrees of active pronation, while bony procedures showed an increase in active pronation with increased variance. Thus, we demonstrate the difference present among patients undergoing soft tissue vs. bony procedures.

The total number of patients assessed across all the studies was 292 (134 male, 133 female, 56 unknown). Number of participants in each study ranged from 4 to 66 patients, with an average of 16.2 patients (standard deviation [SD]: 13.90). Follow-up of patients ranged from 1.3 years up to 8.4 years. Age at time of surgery ranged from 0.3 year old to 14.8 year old. Average age of patients was 7.88 year old (SD = 3.344), and average follow-up was 4.61 (SD = 2.77). More information can be found in Figure 3.

Interventions described in included papers were divided as follows: bony procedures (6 papers), soft tissue procedures (10 papers), or botulinum toxin procedures (2 papers). Bony procedures were made up entirely of osteotomies of the radius, and all had the goal of improving a supination deformity. Soft tissue procedures included tendon and muscle transfers and transpositions, membrane release, release of flexion contracture, tendon lengthening, arthroscopic capsular release, Outerbridge-KashiWagi procedure, muscle transposition, and Steindler flexorplasty/modified Steindler (Table I). The muscles used for transfers or transpositions included biceps, brachioradialis, pectoralis minor, extensor carpi ulnaris, free functioning gracilis, and pronator teres. Functional deficits addressed by soft tissue procedures include elbow flexion restoration, elbow flexion contracture and pronation contracture (Table I).^6^ The most prevalent of all procedures was radius osteotomy (n = 111 patients) discussed in 6 articles, followed by muscle rerouting (n = 86) discussed in 2 articles and finally muscle transfer (n = 54) discussed in 6 articles.

**Table I:** All of the studies included in the paper are shown above with additional data such as their categorization in the paper, aim of the study, and patient information.

Study	Title	Category	Publication year	Number of patients	Follow-up (y)	Age at time of surgery (mean)	Aim	Indications and supple vs. fixed deformity
Allende^2^	Forearm supination deformity after obstetric paralysis.	bony and soft tissue	2004	66	5.35833	6.65833	supination contracture	osteotomies and soft tissue procedures to improve forearm supination, fixed and supple deformities
DeDeugd^10^	Derotational Pronation-producing Osteotomy of the Radius and Biceps Tendon Rerouting for Supination Contractures in Neonatal Brachial Plexus Palsy Patients: A Review of 20 Cases.	bony	2019	20	3	8	supination contracture	combined osteotomy and biceps rerouting, fixed deformity
Hankins^16^	Corrective osteotomies of the radius and ulna for supination contracture of the pediatric and adolescent forearm secondary to neurologic injury.	bony	2006	12	1.33333	11	supination contracture	osteotomy for supination contracture, fixed deformity
Metsaars^28^	Supination Contractures in Brachial Plexus Birth Palsy: Long-Term Upper Limb Function and Recurrence After Forearm Osteotomy or Nonsurgical Treatment.	bony	2017	22	4.6	6	supination contracture	osteotomy for supination contracture, fixed deformity
Metsaars^27^	Biceps Rerouting after Forearm Osteotomy: An Effective Treatment Strategy for Severe Supination Deformity in Obstetric Plexus Palsy.	bony	2017	5	6.8	8	supination contracture	salvage procedure after recurrence of supination deformity after forearm osteotomy, fixed deformity
Van Kooten^20^	Pronating radius osteotomy for supination deformity in children with obstetric brachial plexus palsy.	bony	2008	8	1.9167	9.1	supination contracture	osteotomy for supination contracture, fixed deformity
Jennings^19^	Triplanar Humeral Osteotomy for Restoration of Midline Function in Patients With Brachial Plexus Birth Palsy.	bony	2017	9	2.95	35.4	midline function	Botulinum toxin to triceps to unmask active elbow flexion, supple deformity
Morscher^30^	Onabotulinum toxin type A injection into the triceps unmasks elbow flexion in infant brachial plexus birth palsy: A retrospective observational cohort study.	botulinum toxin	2020	12	6	0.33333	elbow flexion restoration	anterior release and olecranonplasty to improve elbow flexion, fixed deformity
Terzis^44^	Secondary procedures for elbow flexion restoration in late obstetric brachial plexus palsy.	soft tissue	2010	15	8.4	5.4	elbow flexion restoration	pectoralis minor transfer to improve elbow flexion, supple deformity
Gilbert^15^	Obstetrical brachial plexus injuries: late functional results of the Steindler procedure.	soft tissue	2014	27	8.2	4	elbow flexion restoration	augmenting of active supination, supple deformity
Madura^24^	Free functioning gracilis transfer for reanimation of elbow and hand in total traumatic brachial plexopathy in children.	soft tissue	2018	17	6	13.4	elbow flexion restoration	Free functioning muscle transfer for elbow flexion, supple deformity
Costil^8^	Pectoralis minor transfer for elbow flexion restoration in late obstetric brachial plexus palsy.	soft tissue	2018	19	4	6	elbow flexion restoration	augmenting of active supination, supple deformity
Garcia-Lopez^14^	Anterior Release of Elbow Flexion Contractures in Children With Obstetrical Brachial Plexus Lesions	soft tissue	2012	10	3	11.1	elbow flexion contracture	to augment elbow flexion, supple deformity
Price^38^	Result of modified Outerbridge-Kashiwagi procedure for elbow flexion contractures in brachial plexus birth injury.	botulinum toxin	2019	10	3.19167	14.8333	elbow flexion contracture	to release pronation contracture via pronator lengthening/release av lacertus fibrosus, fixed and supple deformity
Amrani^3^	Pronator teres transfer to correct pronation deformity of the forearm after an obstetrical brachial plexus injury	soft tissue	2009	14	1.7	7.6	pronation contracture	to release elbow flexion contracture with biceps lengthening, fixed deformity
Liggio^22^	Outcome of surgical treatment for forearm pronation deformities in children with obstetric brachial plexus injuries	soft tissue	1999	7		8	pronation contracture	to augment elbow flexion, supple deformity
Özkan^33^	'Switch' technique to restore pronation and radial deviation in 17 patients with brachial plexus birth palsy.	soft tissue	2019	17	1.75	8.7	supination and ulnar deviation	to augment elbow extension, supple deformity
Nath^31^	Significant improvement in nerve conduction, arm length, and upper extremity function after intraoperative electrical stimulation, neurolysis, and biceps tendon lengthening in obstetric brachial plexus patients.	soft tissue	2015	7	7	11	bicep tendon flexion contracture	anterior release for elbow flexion contracture, fixed deformity
Ozkan^34^	Brachioradialis transposition for elbow extension in obstetrical brachial plexus palsy.	soft tissue	2005	4	1.33333	7	elbow extension restoration	osteotomy or biceps rerouting to improve pronation, fixed and supple deformity

Thirty-Three different outcome measures were assessed across the 18 papers. Six studies compiled variables describing passive forearm rotation. Five studies collected outcomes regarding active forearm rotation. Passive elbow motion, including flexion and extension, was collected by four studies. Active elbow motion was assessed by five studies. Finally, three studies evaluated wrist motion.

No significant difference (*P* = .6159) was found between the age of patients who received a bony procedure (8.12 year old SD: 1.63) vs. a soft tissue procedure (7.79 year old, SD: 2.76). In addition, no significant differences were found between the length of follow-up of these groups: soft tissue (4.44 years, SD: 2.69) and bony procedures (3.83 years, SD: 2.11) (*P* = .5787). Patients who underwent Botulinum toxin injection comprised only 2 studies with a total of 22 patients in this review, and thus were excluded and were not analyzed due to lack of substantial data.

An analysis of subgroup using itemized data (3 studies describing bony procedures and 10 studies describing soft tissue procedures) was performed to compare preoperative and postoperative range of motion outcomes. For soft tissue procedures, a statistically significant increase was found for active elbow flexion (*P* < .01), passive supination (*P* < .05), and active supination (*P* < .01) postoperatively (Table II). For bony procedures, where the aim was to correct a fixed deformity, a statistically significant reduction was found for passive supination (*P* < .01) and active supination (*P* < .01), and a statistically significant increase was found in active pronation (*P* < .01) (Table II). These results reflect the *P* values from the paired Wilcoxon test. Significance was also found in the same categories using the paired t-test to confirm our results.

**Table II:** Outcome measures of subgroup with itemized data comparing preoperative and postoperative values is shown above along with *P* values for paired t-tests and paired Wilcoxon tests.

Procedure type	Outcome measure	Preop mean (SD)	Postop mean (SD)	Paired t-test *P* value	Paired Wilcoxon test *P* value
Bony	Passive supination	88 (7.5)	58 (26.6)	.002778	.005859
	Active supination	67 (24.5)	23 (28.0)	.006824	.009766
	Active pronation	−14 (15.6)	33 (26.7)	7.94e−5	.0004883
Soft tissue	Active elbow flexion	56 (57.8)	114 (30.2)	6.529e−12	3.001e−11
	Elbow extension	13 (39.6)	9 (34.8)	.2382	.2238
	Passive supination	33 (25.5)	76 (7.5)	.003054	.03125
	Active supination	3 (4.8)	66 (19.5)	3.235e−12	9.537e−7
	Active pronation	80 (0)	73 (18.9)	.2559	1

*SD*, standard deviation.

To compare bony and soft tissue procedures, the unpaired Wilcoxon test was used to assess differences in preoperative and postoperative values between the groups. Preoperatively, bony procedure patients were found to have statistically significantly higher values for passive and active supination (*P* = 8.668e−6, *P* = 8.785e−11). However, postoperatively, soft tissue procedure patients were found to have higher values for active supination (*P* = 5.43e−5), again mirroring the differences in indications for surgery between bony procedures being used for fixed deformities and soft tissue procedures for a mix of supple and fixed deformities. No significant difference was found postoperatively between the two groups for passive supination. Active pronation was found to have a statistically significantly higher value for soft tissue procedures both preoperative and postoperatively (*P* = 1.29e−5, *P* = .0006837) (Table III).

**Table III:** Outcome measures between bony vs. soft tissue procedures preoperative and postoperative values are compared above with *P* values for unpaired Wilcoxon tests presented.

Subgroup	Outcome measure	Subgroup with the higher value	Unpaired Wilcoxon test *P* value
Preoperative	Passive supination	Bony	8.668e−6
	Active supination	Bony	8.785e−11
	Active pronation	Soft tissue	1.29e−5
Postoperative	Passive supination	No difference	.2273
	Active supination	Soft tissue	5.435e−5
	Active pronation	Soft tissue	.0006837

**Figure 4:**
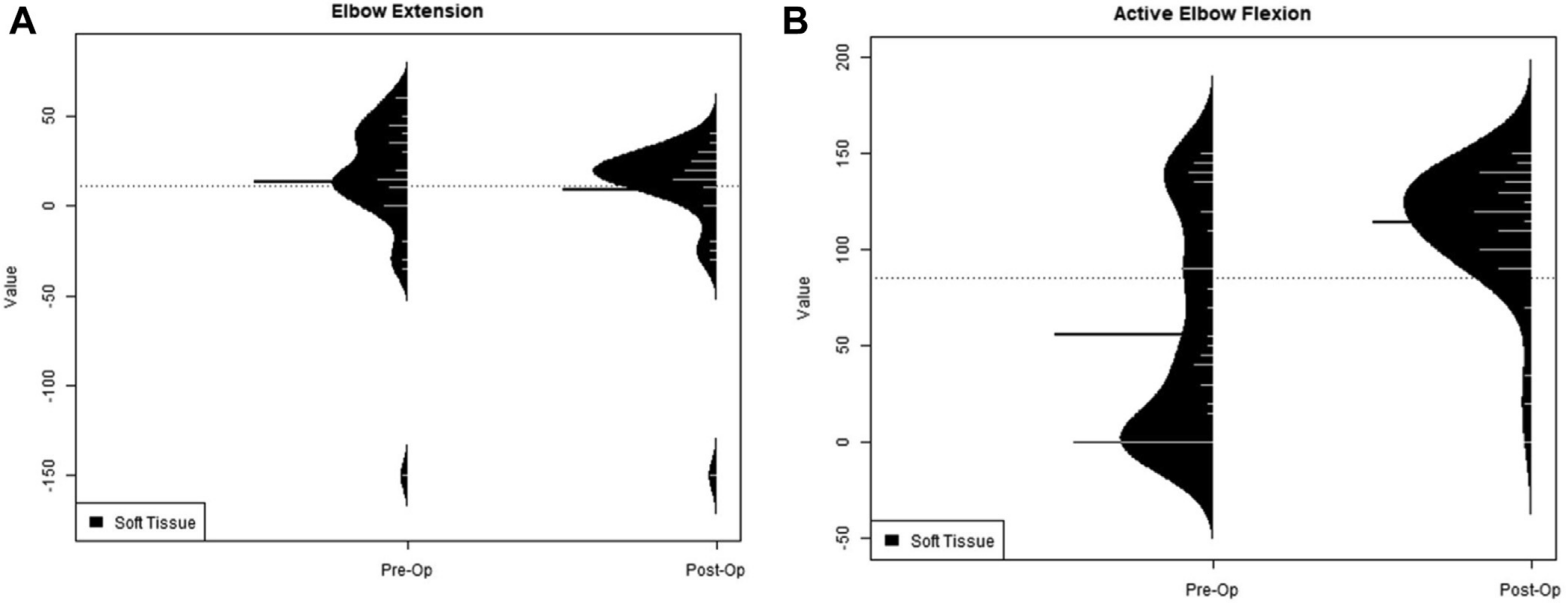
Soft tissue procedures also described active elbow extension (**A**) and flexion (**B**). This depicts a reduction in variance with an increase in degrees of motion for active flexion with a reduction in variance with a similar degree of motion for active extension.

A comparison of the distributions of the itemized preoperative and postoperative data for active and passive supination, active pronation, and elbow flexion and extension was also completed using the KS test (Figs. 3 and 4). The distribution comparison for forearm rotation revealed differences between outcomes of bony and soft tissue procedures. For soft tissue procedures, there was a narrowed variance and an increase in range of motion postoperatively with regards to active and passive supination, while preoperative and postoperative active pronation was grossly unchanged and located in good functional range. For bony procedures, however, the postoperative variance in active and passive supination was wide, suggesting a broader range of outcomes after osteotomies. When active pronation was examined for bony procedures, the increased range of pronation was seen with similarly high variance, suggesting a range of possible outcomes after osteotomies. Only soft tissue procedures reported outcomes for the elbow’s range of motion. For elbow flexion, the variance decreased postoperatively leading to improved active flexion within a useful range. Elbow extension was found to have a somewhat decreased variance with a similar range of motion postoperatively (Fig. 4).

## Discussion

Our review found 18 studies describing outcomes of secondary procedures for forearm and elbow for NBPP patients. We found that a range of procedures are performed for this group of patients, including bony, soft tissue and botulinum toxin procedures, with different aims and reporting diverse outcome measures. While all procedures report improvement for the intended aim, in the subgroup analysis of studies providing itemized patient data we have identified differences between bony and soft tissue procedures. Soft tissue procedures were found to have a wider variety of aims including restoring forearm supination and elbow flexion. Bony procedures were comprised entirely of radius osteotomies, with the only goal being resolution of a supination contracture. Overall, we found that bony procedures and soft tissue procedures were performed for patients of similar age and follow-up time. Using the KS test, we found that variance of most distributions of soft tissue procedures’ outcomes decreased while the variance of all bony procedures’ outcomes increased, suggesting that patients undergoing radius osteotomies obtain a wider range of outcomes, with some of them risking results outside functional range of motion in the forearm. This risk of poor functional outcome, with limited pronation and supination should be included in consent for surgery and communicated to the patient and family.

Soft tissue procedures mostly aimed to restore elbow flexion, and a smaller number of the studies aimed to restore active supination of the forearm. Most soft tissue procedures are indicated to address supple rather than fixed deformities, and this holds true for most papers included in this study. Statistically significant differences were found in between preoperative and postoperative active flexion with postoperative values yielding higher results, showing that various soft tissue procedures including muscle transfers and transpositions and tendon transfers are reliable in improving active elbow flexion. In addition, there was no difference found between preoperative and postoperative active extension demonstrating that these procedures do not lead to loss of the opposing function. In our literature review, we found several studies completed between 2002 and 2015 assessed NBPP patients who were operated on as adults. They found that different combinations of muscle transfers were able to achieve restoration of adequate elbow flexion, without loss of extension in this patient population even as adults, which mirrors our findings in this study.^5^^,^^12^ In addition, despite many of these studies having flexion as their stated aim, our data shows that not only were these patients able to restore some degree of flexion, but they were also able to regain more range of motion in other planes such as supination. Significant differences were found between preoperative and postoperative active supination as well, postoperative being higher values, with no difference in active pronation. This supports the idea that soft tissue procedures are more fit for patients with more diffuse range of motion deformities, and they are more tailored to the losses presented by each patient.^5^ Literature supports that these procedures have the capacity to affect several muscles and result in functional gains which can impact the entire upper limb from shoulder to the hand.^26^ Therefore, these procedures are valuable for the patient population targeting the recovery of active flexion and supination while maintaining pronation and extension.

The only procedure completed for a supination contracture was an osteotomy. No other procedure had more patients undergoing it than those who underwent an osteotomy. This is due to supination contractures making up 7% of children with brachial plexus birth injuries.^25^ In our results patients had a high degree of active and passive supination preoperatively. Both were found to be statistically significantly decreased postoperatively, thus showing the achievement of the procedure’s goal. A statistically significant increase was also in pronation postoperatively, thus restoring some range of motion that these patients may not have previously had. These results may simply mirror indications for surgery in this patient population. A study described the usage of osteotomies for patients across multiple pathologies including NBPP patients found that the procedure achieves a large degree of correction of the supination contracture.^7^ Interestingly, when comparing postoperative passive supination between patients who underwent bony vs. soft tissue procedures, no significant difference was found. These findings further highlight that patients who undergo osteotomy return to more normal passive supination.

The KS test is an innovative statistical technique that allowed us to compare the distributions and variances of the outcomes in the subgroups. Literature has established the utility of this tool in for distribution analysis,^48^^,^^49^ and this was done in a study describing total knee arthroplasty and, in another study describing postsurgical pain.^43^^,^^50^ In our study, we found that bony procedures led to a widened variance postoperatively. This may imply less consistent results in bony procedures as patients widely varied in the degree of resolution, they achieved with regard to their supination contracture. Thus, this may indicate the need for other procedures to improve this deformity with more consistent results. With a positively skewed active pronation distribution, patients with this supination contracture did tend to regain some active pronation postoperatively. This signifies a gain of function in a plane these patients may not have previously had. Soft tissue procedures led to a narrowed variance postoperatively across most outcomes including passive supination, active elbow flexion, and active elbow extension. This indicates that soft tissue procedures lead to more consistent, positive outcomes for patients who underwent them. It also implies a gain of function in the desired range of motion without loss of antagonistic function, shown by a gain of flexion without a loss of extension.

Overall, this review could not answer the question of whether gains assessed were sustained following growth spurts depending on the procedure completed^18^ as follow-up times varied widely and may not have included the patients during or after they took place. With regards to elbow function, a study found that both primary and secondary surgical patients sustained better flexion and extension at the 10-year mark than did those that did not undergo surgery.^18^ However, regarding forearm function, specifically pronation and supination, patients who underwent surgery resulted in less active motion than those who did not undergo surgery.^18^ Moreover, patients undergoing these procedures should be followed for more extended periods to better understand the long-term benefits of these surgeries.

Several limitations exist in the review of these articles, as included studies reported a variety of outcomes, procedures, and surgical goals, which lead to difficulty in statistical analysis. A consensus survey from iPluto discusses these present issues including the usage of many outcome measures, the various follow-up and the various ages. This study found that initial evaluations should take place at 1, 3, 6, and 9 months of age and should assess external rotation, abduction, elbow flexion, wrist extension, and finger flexion and extension. With regards to treatment outcomes, patients should be assessed at 1/3/5/7 years and once again at 15 years of age. For passive range of motion, external rotation, abduction, and elbow extension should be included in the data set. For active range of motion, they found that external rotation, abduction, elbow flexion and extension, wrist extension, and finger flexion and extension should be collected.^35^ However, despite these guidelines, we found that many of the papers did not collect all these variables at these time marks. Although grouping the papers allowed us to identify some common goals within groups, subgroup analysis remained challenging due to heterogenous outcome measures.^40^ Further research is also indicated with regards to forearm and elbow secondary surgeries as only 18 full-text articles fulfilled our inclusion criteria, and studies regarding long-term benefits are warranted as few studies have followed these patients for more than 5 years. Overall, more research and standardization of outcome collection is needed to provide more insight into the efficacy of these procedures.

## Conclusion

There is a variety of surgeries that can be useful to improve forearm and elbow deformities in NBPP patients. Both bony and soft tissue procedures are performed on average at age 7.8 year old. This study demonstrates that both procedure subgroups were successful not only in improving the range of motion they aimed for but also in maintaining moderate antagonist function. Soft tissue procedures lead overall to a narrower distribution of outcomes, while radius osteotomies result in a wider distribution of functional outcomes. More studies are needed on the topic of forearm and elbow secondary surgical procedures to fully assess the efficacy and long-term benefits of these interventions.

## Disclaimers:

Funding: No funding was disclosed by the authors.

Conflicts of interest: The authors, their immediate families, and any research foundation with which they are affiliated have not received any financial payments or other benefits from any commercial entity related to the subject of this article.
